# Supplementary material for: Causal Relationship between Aging and Anorexia Nervosa: A White-Matter-Microstructure-Mediated Mendelian Randomization Analysis
Source: Biomedicines. 2024 Aug 16;12(8):1874. doi: 10.3390/biomedicines12081874 (PMC11351342; doi:10.3390/biomedicines12081874)
Supplement: Supplementary file 1 [file biomedicines-12-01874-s001.zip › Supplementary Figure/Figure S4/Singe SNP plot of MR analysis for TL to ubm-b-1552.pdf]

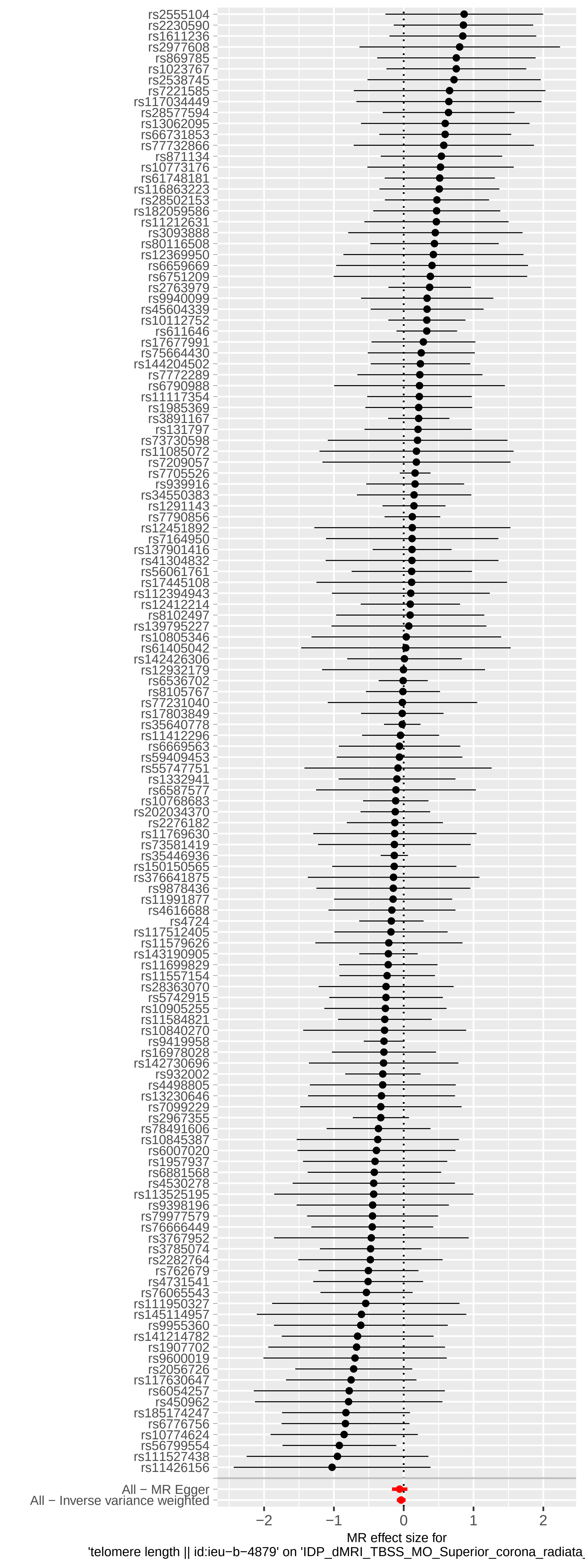
rs2555104
rs2230590
rs1611236
rs2977608
rs869785
rs1023767
rs2538745
rs7221585
rs117034449
rs28577594
rs13062095
rs66731853
rs77732866
rs871134
rs10773176
rs61748181
rs116863223
rs28502153
rs182059586
rs11212631
rs3093888
rs80116508
rs12369950
rs6659669
rs6751209
rs2763979
rs9940099
rs45604339
rs10112752
rs611646
rs17677991
rs75664430
rs144204502
rs7772289
rs6790988
rs11117354
rs1985369
rs3891167
rs131797
rs73730598
rs11085072
rs7209057
rs7705526
rs939916
rs34550383
rs1291143
rs7790856
rs12451892
rs7164950
rs137901416
rs41304832
rs56061761
rs17445108
rs112394943
rs12412214
rs8102497
rs139795227
rs10805346
rs61405042
rs142426306
rs12932179
rs6536702
rs8105767
rs77231040
rs17803849
rs35640778
rs11412296
rs6669563
rs59409453
rs55747751
rs1332941
rs6587577
rs10768683
rs202034370
rs2276182
rs11769630
rs73581419
rs35446936
rs150150565
rs376641875
rs9878436
rs11991877
rs4616688
rs4724
rs117512405
rs11579626
rs143190905
rs11699829
rs11557154
rs28363070
rs5742915
rs10905255
rs11584821
rs10840270
rs9419958
rs16978028
rs142730696
rs932002
rs4498805
rs13230646
rs7099229
rs2967355
rs78491606
rs10845387
rs6007020
rs1957937
rs6881568
rs4530278
rs113525195
rs9398196
rs79977579
rs76666449
rs3767952
rs3785074
rs2282764
rs762679
rs4731541
rs76065543
rs111950327
rs145114957
rs9955360
rs141214782
rs1907702
rs9600019
rs2056726
rs117630647
rs6054257
rs450962
rs185174247
rs6776756
rs10774624
rs56799554
rs111527438
rs11426156
All − MR Egger
All − Inverse variance weighted
−2
−1
0
1
2
MR effect size for
'telomere length || id:ieu−b−4879' on 'IDP_dMRI_TBSS_MO_Superior_corona_radiata_
